# Characterization and functional analysis of a slow-cycling subpopulation in colorectal cancer enriched by cell cycle inducer combined chemotherapy

**DOI:** 10.18632/oncotarget.19638

**Published:** 2017-07-26

**Authors:** Feng-Hua Wu, Lei Mu, Xiao-Lan Li, Yi-Bing Hu, Hui Liu, Lin-Tao Han, Jian-Ping Gong

**Affiliations:** ^1^ Cancer Research Institution, Tongji Hospital, Tongji Medical College, Huazhong University of Science and Technology, Wuhan 430032, People’s Republic of China; ^2^ Department of Physiology, Hubei University of Chinese Medcine, Wuhan 430065, People’s Republic of China

**Keywords:** slow-cycling tumor cells, cancer stem cell, DC-CIK, adoptive transfer, tumor dormancy

## Abstract

The concept of cancer stem cells has been proposed in various malignancies including colorectal cancer. Recent studies show direct evidence for quiescence slow-cycling cells playing a role in cancer stem cells. There exists an urgent need to isolate and better characterize these slow-cycling cells. In this study, we developed a new model to enrich slow-cycling tumor cells using cell-cycle inducer combined with cell cycle-dependent chemotherapy *in vitro* and *in vivo*. Our results show that Short-term exposure of colorectal cancer cells to chemotherapy combined with cell-cycle inducer enriches for a cell-cycle quiescent tumor cell population. Specifically, these slow-cycling tumor cells exhibit increased chemotherapy resistance *in vitro* and tumorigenicity in *vivo*. Notably, these cells are stem-cell like and participate in metastatic dormancy. Further exploration indicates that slow-cycling colorectal cancer cells in our model are less sensitive to cytokine-induced-killer cell mediated cytotoxic killing *in vivo* and *in vitro*. Collectively, our cell cycle inducer combined chemotherapy exposure model enriches for a slow-cycling, dormant, chemo-resistant tumor cell sub-population that are resistant to cytokine induced killer cell based immunotherapy. Studying unique signaling pathways in dormant tumor cells enriched by cell cycle inducer combined chemotherapy treatment is expected to identify novel therapeutic targets for preventing tumor recurrence.

## INTRODUCTION

In the majority of cancer cases, mortality is caused by metastasis or recurrence, with only 10% being caused by the primary tumor [1]. Based on the concept of cancer stem cells (CSCs), claims are made that the ability of a tumor to grow and propagate may depend on small subsets of cells exhibiting increased self renewal and tumor initiating potential [2–4]. Surface antigens such as CD24, CD44, CD133 or ALDH activity are used as CSC markers gradually. Unfortunately, the use of CSC markers has not been without controversy [5, 6]. Recent studies show direct evidence for quiescence slow-cycling cells playing a role in CSCs [7, 8]. The ability to isolate and better characterize these slow-cycling cancer cells will be a key component of future therapies that better target CSCs [9].

An alternative therapeutic strategy to eliminate quiescent tumor cells was to evicting them from their safe havens prior to cytotoxic therapy administration [10]. Based on the above, Saito et al [11] showed that cytokine treatment induces entry into cell cycle and enhances elimination of human primary acute myelocytic leukemia (AML) stem cells combined with cycle-dependent chemotherapy. But the characteristics of the remaining cells after above cytokine-combined chemotherapy are not well studied and it is unclear whether these characteristics contribute to tumor recurrence.

Traditional chemotherapies like 5-fluorouracil (5-FU) and Oxaliplatin require active cycling cells to trigger cell death [12, 13], leaving a slow-cycling tumor cell sub-population which are less likely to be susceptible to these drugs [14]. Colorectal cancer is characterized by overexpressing epidermal growth factor receptor (EGFR) [15]. EGFR signal pathway promotes tumor proliferation in correlated with cell cycle progression [16]. In present study, we developed a model to enrich slow-cycling tumor cells (which were named as SCCs as follow) by delivery EGF as a cell cycle inducer combined short-term chemotherapy. We demonstrated that these slow-cycling cells were more tumorigenic and more resistant to traditional chemotherapies. Further, we found that these cells were the source of tumor relapse and metastasis, and are thus an obstacle to therapy.

The transfusion of lymphocytes, such as adoptive T cell therapy, is being tested for the treatment of cancer [17]. Cytokine induced killer (CIK) cells are a group of heterogeneous cells stimulated by multiple cytokines with anti-tumor activity. Dendritic cells (DC) are antigen presenting cells (APCs) that play an important role in antigen specific cytotoxic T lymphocyte response. Dendritic cells co-cultured with cytokine induced killer cells (herein after referred to as DC-CIK cells) should be an antigen specific treatment and have been reported to be applied for various tumor treatments [18–20]. Here, we investigated DC-CIK based immunotherapy on SCCs. We demonstrated that the cytotoxicity of DC-CIK cells on these slow-cycling tumor cells was much lower compared with that on control tumor cells, suggesting that they may be involved in immune-mediated dormancy as well as cellular dormancy. Our present study provides an opportunity for targeting cancer in novel immune treatments.

## RESULTS

### SCCs enriched by cell cycle inducer combined chemotherapy *in vitro* are more approximate to cell-cycle quiescence

**Figure 1:**
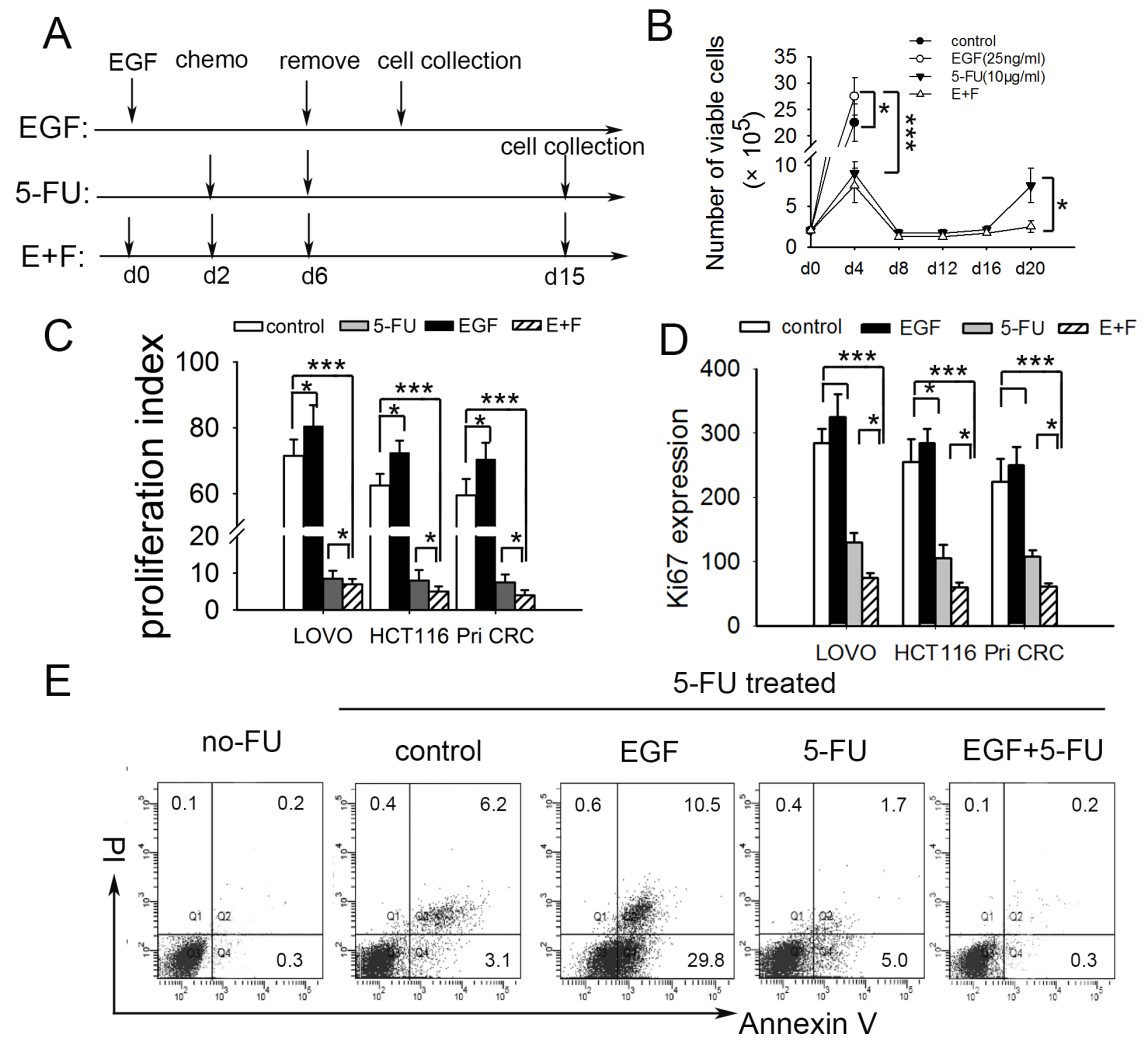
SCCs enriched by cell cycle inducer combined chemotherapy are more approximate to cell-cycle quiescent cells **(A)** Schematic of experimental slow-cycling cell model *in vitro*. For E+F group, human colorectal tumor cells were incubated with cell cycle inducer (recombinant EGF) for 2 d, after which chemotherapy (5-FU) was added for 4 d. Then chemotherapy was removed and new media added. The residual tumor cells were harvested on day 15 for further experiments. For EGF or 5-FU alone group, tumor cells received EGF or 5-FU treatment alone according to the schematic. **(B)** Cell-cycle inducer makes colorectal cancer cells more sensitive to chemotherapy. Colorectal tumor cells were treated as above. Cell proliferation was measured by counting the number of viable cells at indicated time point. The growth curve of LoVo cells was showed as representation. **(C)** Cell cycle inducer combined chemotherapy enriches slow-cycling tumor cells. Colorectal tumor cell lines (LoVo and HCT116) or primary colorectal cancer cells from patient (Pri CRC) were labled with proliferation marker CFSE and treated as above for 8d away from light. The proliferation of cells was analyzed by flow cytometry, and proliferation index was calculated. **(D)** Ki67 was low expressed in slow-cycling tumor cells. Tumor cells treated as above were collected on day 15. Ki67 expression was analyzed by flow cytometry. Ki67^+^ expression index was calculated by using the formula: mean fluorescence × percentage of Ki67^+^ cells. **(E)** Slow-cycling tumor cells were chemoresistant. Harvested LoVo cells after different treatment as above were exposed to second 5-FU for 24 h. 24 h after 5-FU removing, apoptosis of cells was analyzed by flow cytometry. Data are representative of three independent experiments with triplicate samples in each group. *P* values, *P<0.05; ***P<0.001.

It is reported that short-term exposure of tumor cells to chemotherapy enriches for a slow-cycling, chemo-resistant tumor cell sub-population [14]. Given that cell cycle inducer makes tumor cells more sensitive to chemotherapy [11], we wondered whether cell cycle inducer combined with chemotherapy may enrich cell-cycle quiescent cells. To test this, we exposed primary colorectal tumor cells from patient (Pri CRC) and colorectal cancer cell lines to above combined application (Figure 1A). EGF was used as cell-cycle inducer since EGFR signaling promoted cell-cycle progression in colorectal cancer [16]. The result showed that cell-cycle inducer promoted the proliferation of colorectal tumor cells and made them more sensitive to chemotherapy by microscope counts in LoVo cells (Figure 1B). The similar results were found in other cancer cells (data not shown). MAPK signaling downstream of EGFR controls colorectal tumor cell proliferation [21]. Immunoblot analysis showed a declined expression of EGFR in SCCs. Furthermore, the phosphorylation of EFGR and ERK1/2 in SCCs were weaker than that in control cells upon EGF treatment (Supplementary Figure 1A). Previous studies showed that EGFR signal is down regulated in quiescent cancer stem cells [22]. The downregulation of EGFR signal may contribute to quiescence maintaining of SCCs in our model. These studies implied that cell cycle inducer combined chemotherapy enriched for a slow-cycling tumor cell subgroup more approximate to cell-cycle quiescence *in vitro*.

We further performed proliferation assay by CFSE staining to quantitatively evaluate proliferation of residual cells following above treatment. As shown in Figure 1C, the proliferation of residual cancer cells after combined treatment was much lower than that of control and EGF groups, and lower than that of 5-FU group slightly. The expressions of Ki67, which is commonly used to detect and quantify proliferating cells [23], were investigated meanwhile. The results showed that Ki67 expression in SCCs was lower than that of the other three groups (Figure 1D). Besides these, the residual cells enriched by cell cycle inducer combined chemotherapy were more resistant to a second 5-FU treatment (Figure 1E, Supplementary Figure 1B), which is consistent with the previous prediction that dormant tumor cells are an obstacle to traditional chemotherapy [24]. Furthermore, we conducted anokis assay to access cell death of tumor cells when detached from surrounding extracellular matrix. SCCs showed higher anoikis resistance capacity (Supplementary Figure 2). These imply that SCCs in our model were chemotherapy and anoikis resistant.

### SCCs enriched by cell cycle inducer combined chemotherapy exhibit increased tumorigenicity *in vivo*

**Figure 2:**
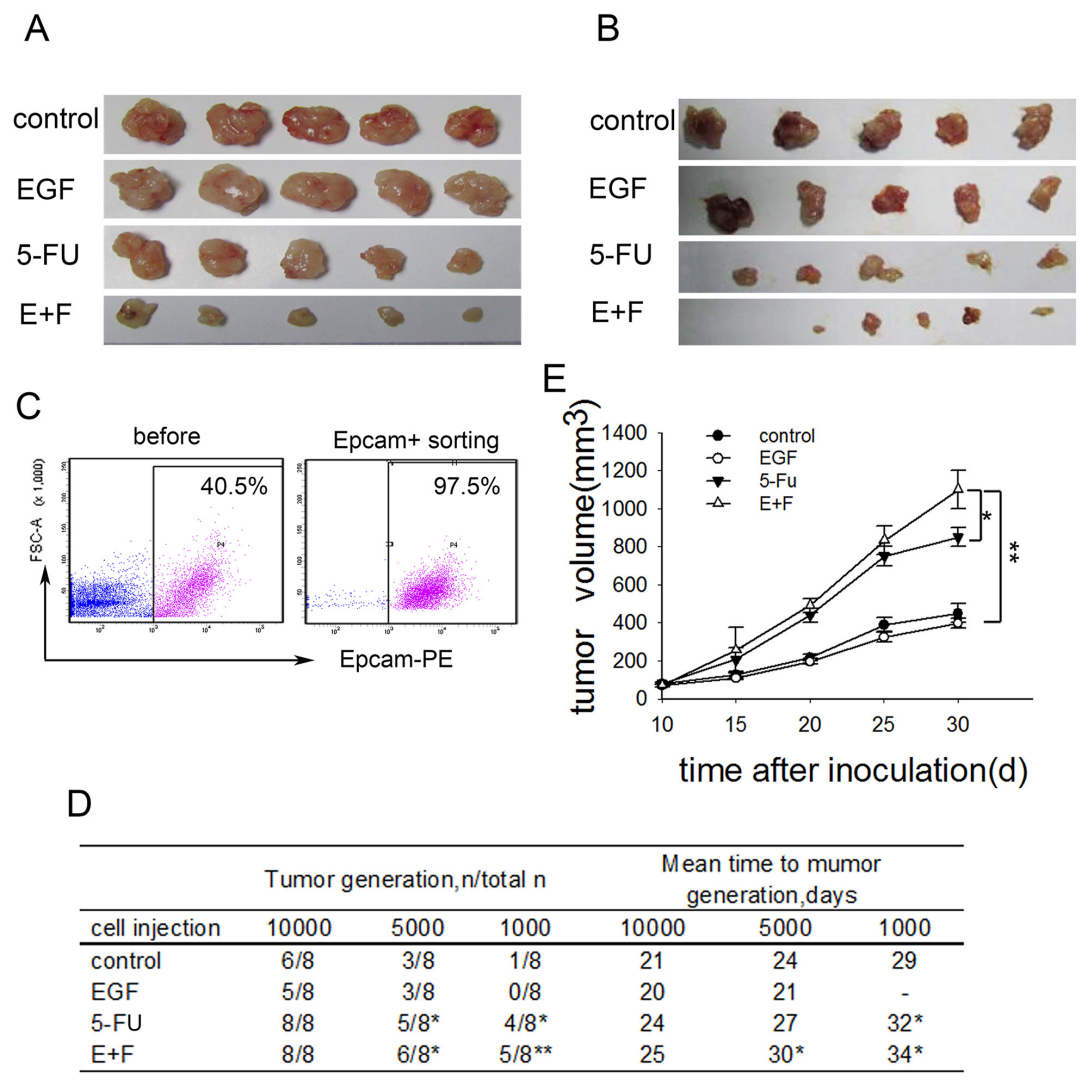
SCCs enriched by cell cycle inducer combined chemotherapy exhibit increased tumorigenicity *in vivo* **(A)** Effect of combined treatment on tumor growth. Mice were subcutaneously inoculated with LoVo cells, and then received administration of EGF alone, 5-FU alone or combined treatment. Tumors (n=5 in each group) were dissected on day 30 after tumor inoculation. Representative tumor tissues were presented. **(B)** Nude mice subcutaneously inoculated with tumor cells from primary colorectal tumors received the same treatment as above. Representative tumor tissues were presented. **(C)** and **(D)** Tumor-generation assay using injection of tumor cells in a gradient dose. Tumor tissues from mice challenged with LoVo cells after above treatments were dissected and digested. Single cell suspensions were prepared. Tumor cells were enriched by EpCAM^+^ FACS sorting from single cell suspensions for all the cell groups (C) and subcutaneously inoculated into Nude mice again in a gradient dose. Tumor generation was observed (D). The *t-test* was carried out to determine the difference between experimental group and control group in three experiments. *P<0.05; **P<0.01. **(E)** Tumor growth *in vivo*. Tumors cells collected as above were subcutaneously inoculated into Nude mice at a high dose. Tumor growth was monitored at indicated time. Data are pooled from three independent experiments with a total of five mice in each group. Data are represented as mean ± SD. *P* values, *P<0.05, **P<0.01.

To investigate how cell cycle inducer delivery combined chemotherapy affect tumor growth *in vivo*, we performed transplantation tumor model by injecting LoVo cells and primary colorectal tumor cells subcutaneously into the flank of Nude mice. The tumor-bearing mice were treated with the respective regimens according to the treatment schematic shown as Supplementary Figure 3. In consist with what we had observed in *in vitro* experiments, tumors in mice treated with cell cycle inducer combined chemotherapy were clearly reduced compared with those in other groups (Figure 2A and 2B). To assess the tumorigenicity of transplantation tumor, we further inoculated LoVo cells derived from above xenograft tumors into Nude mice again. To do this, xenograft tumors were digested to obtain single cell suspensions. Tumor cells were enriched by EpCAM^+^ (epithelial cell adhesion molecule) FACS sorting from single cell suspensions (Figure 2C) and then inoculated to Nude mice in a gradient dose. We found that tumor cells derived from xenograft tumors after combined treatment exhibited the highest tumorigenic potential among the four groups, whereas, the average number of days of tumor generation was prolonged compared with that of the other three groups (Figure 2D). Moreover, transplanted tumor cells grow much faster when inoculated into Nude mice with a high dose (Figure 2E). These findings suggested that delivery of cell cycle inducer combined chemotherapy *in vivo* enriched SCCs with advanced tumorigenic potential.

### SCCs enriched by cell cycle inducer combined chemotherapy are stem-cell like and participate in metastatic dormancy

**Figure 3:**
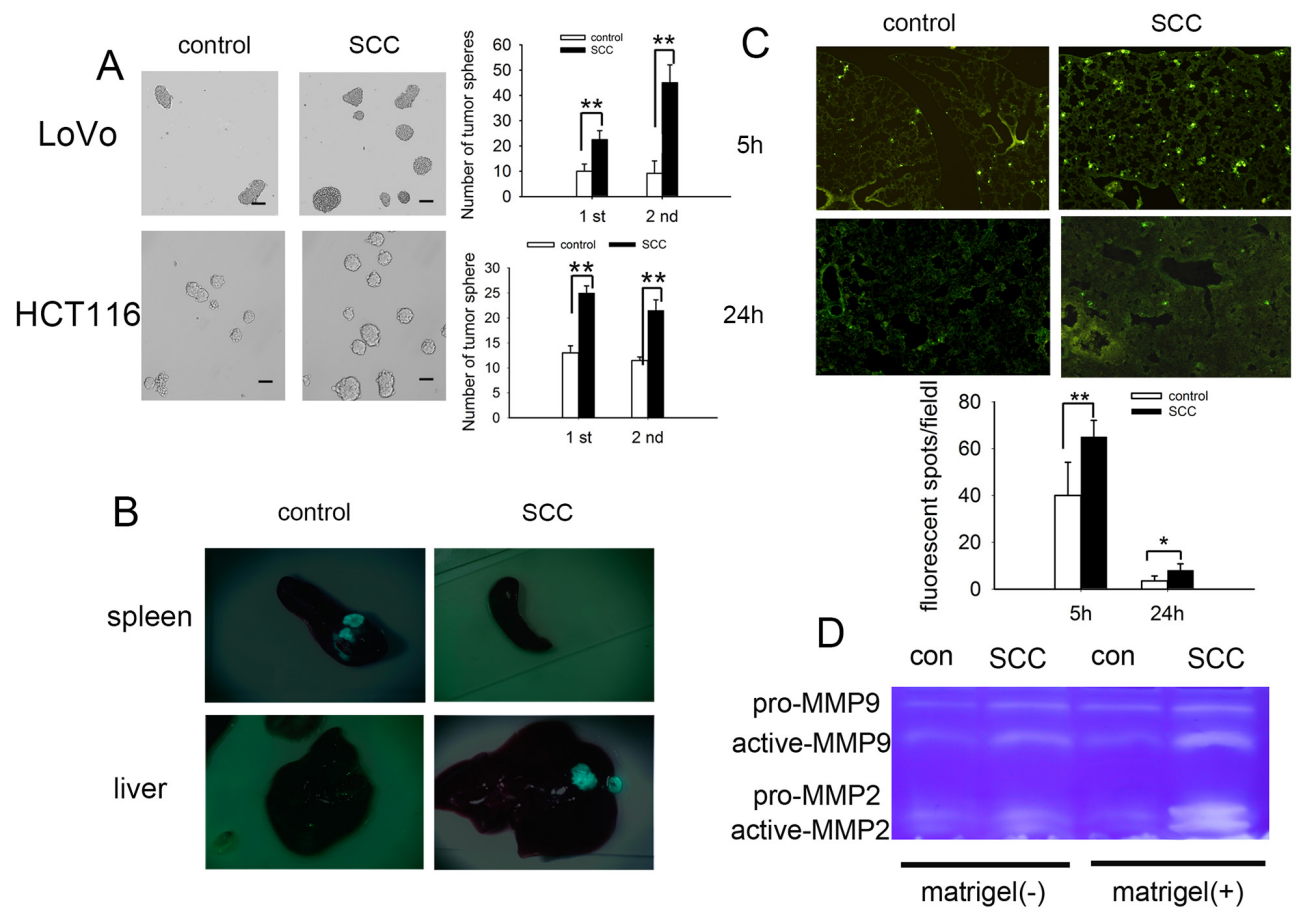
SCCs enriched by cell cycle inducer combined chemotherapy are stem-cell like and participate in metastatic dormancy **(A)** Tumorsphere culture from control tumor cells (HCT116 and LoVo) and SCCs. Much more tumorspheres were obtained from SCCs for the first passage and the SCC tumorspheres can be more efficiently passaged and expanded (the second passage). Scale bars represent 50 μm. **(B)** Tumor metastasis to liver by intra-spleen injection. The mice were inoculated by intraspleen injection of control LoVo-GFP cells or SCCs-GFP. On day 35 after inoculation, mice were sacrificed and tumor nodes on both spleen and liver were observed. Representative tumor tissues were presented. **(C)** Tumor cell retention in lung by intravenous injection. Control LoVo cells or SCCs were labeled with CFSE and injected into mice via tail vein. Mice were killed 5 h or 24 h after the i.v. injection of tumor cells. The CFSE-labeled tumor cells in frozen sections were visualized and counted by fluorescence microscopy. **(D)** Production of MMP-9 and MMP-2 in the presence or absence of ECM molecules (matrigel). Control LoVo cells or SCCs were cultured in the presence or absence of matrigel. MMP-9 and MMP-2 in supernatants were detected by Zymography assay. Data are representative of three independent experiments (A, B, D) or pooled from three independent experiments with a total of five mice in each group (C). *P* values, *P<0.05, **P<0.01.

On the base of above studies that SCCs in our model regained proliferation ability, resembling tumor recurrence *in vivo* (Figure 2A-2D), we further investigated whether such a repopulating capability may go along with increased tumorigenicity. We performed a tumorsphere assay by seeding 200 tumor cells in 24 well plates. The results showed that SCCs generated significantly more tumorspheres than control tumor cells. Moreover, we found that the SCCs tumorspheres could be passaged more efficiently than that of control tumor spheres (Figure 3A). The typical stem cell markers such as CD133, CD44 and LGR5 were also high expressed on SCCs (Supplementary Figure 4). CD133, a predictor of early recurrence in colorectal cancer [25], was significantly over expressed on SCCs.

Previous studies had shown that quiescence sorted by dye retention assays played a role in CSCs [7, 26, 27], and predisposed for the development of metastasis [28]. We then investigated whether SCCs participated in metastasis initiation by using a model of liver metastasis of splenic colorectal cancer. When LoVo-GFP cells were inoculated to the spleen of mouse, the tumorous nodes formed in spleen 30 days after inoculation, but not in the liver. Nevertheless, metastasis of tumor cells from spleen to liver was observed in SCCs-GFP groups in spite that the sizes of tumor nodes in spleen were significantly smaller than those in control groups (Figure 3B). Once in circulation, cancer cells must overcome a series of stress events to extravasate and invade the parenchyma of a distant tissue. To assess tumor cell retention in lung under dynamic flow conditions, we injected CFSE-labeled LoVo cells into nude mice via tail vein. Cell arrest and extravasation in the lung were assessed. The fluorescent spots in lung tissues were significantly increased both 5 h and 24 h after i.v. injection SCCs, suggesting that cell arrest (5 h) and extravasation (24 h) of SCCs were higher than that of control cells (Figure 3C). The microenvironment is critical for the switch from a dormant state to an active one [29]. Here we analyzed the production of MMPs in tumor cells with or without ECM molecules (Matrigel) stimulation. The production of MMPs in response to ECM molecules (Matrigel) was increased in spite of their expression being unchanged in the absence of Matrigel in SCCs group (Figure 3D). Taken together, these data suggested that SCCs enrich by cell cycle inducer combined chemotherapy were stem-cell like and participated in metastatic dormancy.

### SCCs enriched by cell cycle inducer combined chemotherapy are more resistant to DC-CIK-mediated cytotoxic killing *in vitro*

If dormant tumor cells can persist in the presence of an efficient immune response and can induce frequent relapses, they must counteract the immune effectors or resist their cytotoxic effects [30]. Here we investigated whether SCCs in our model were resistant to immunotherapy based on DC-CIK cells.

Immune cells were prepared from peripheral blood mononuclear cells. DCs and CIK cells showed cluster-like growth (Supplementary Figure 5A). After incubation for 1, 6 and 7 days, expressions of HLA-DR, CD80 and CD86 on DCs were analyzed to confirm DCs maturation by flow cytometry (Supplementary Figure 5B). CIK cells were cultures for 7 days individually and then co-cultured with matured DCs for 2 days. Phenotypes of CIK cells were also detected (Supplementary Figure 5C). CIK cells co-cultured with DCs for 2 days were then used as the effector cells.

**Figure 4:**
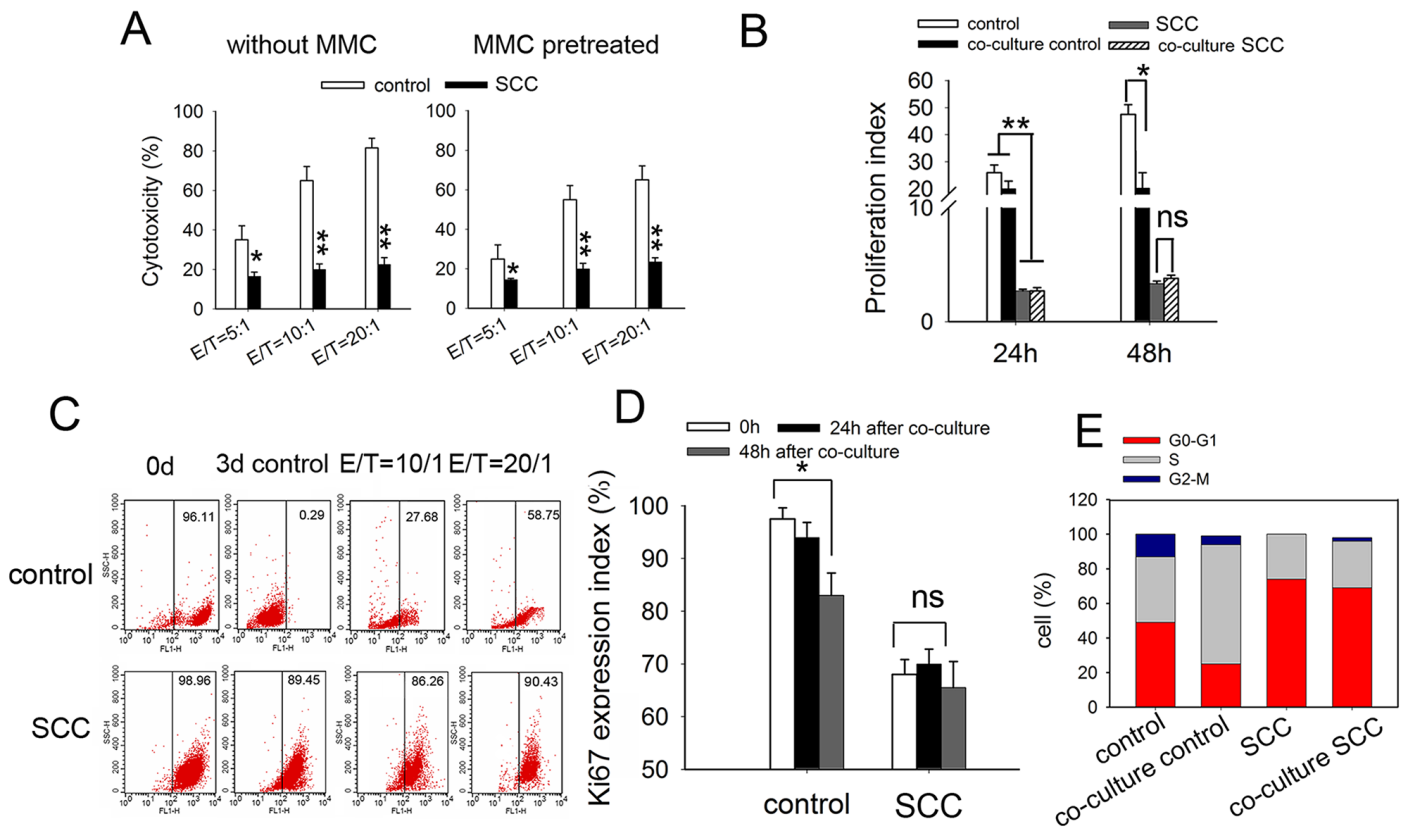
SCCs enriched by cycle-inducing combined chemotherapy are more resistant to DC-CIK-mediated cytotoxic killing *in vitro* **(A)** Cytotoxicity assay of DC-CIK cells on indicated tumor cell. SCCs or control LoVo cells with (right) or without (left) MMC pretreatment were co-cultured with DC-CIK cells for 48h. The cytotoxicity was measured by CCK-8 assay. **(B)** Tumor cells were stained with CFSE and co-cultured with DC-CIK cells with a Transwell co-culture system at E/T radio of 20:1 for 24 h or 48 h. The proliferation of tumor cells was analyzed by flow cytometry, and proliferation index was calculated. **(C)** Lable-retaining tumor cells were enriched *in vivo* after treatment with DC-CIK cells. The CFSE-labled tumor cells were co-cultured with DC-CIK cells for 3 d. Lable-retaining tumor cells were analyzed by flow cytometry. Ki67 expression **(D)** and cell cycle distribution **(E)** of these tumor cells 3 days after co-cultured with DC-CIK cells were analyzed by flow cytometry. Data are pooled from three independent experiments with triplicate samples in each group. *P* values, *P<0.05, **P<0.01.

We first compared the antitumor effect of DC-CIK cells on SCCs with that on control LoVo cells by *in vitro* cytotoxicity assay. The results showed that SCCs were less susceptible to DC-CIK killing, with decreasing levels of cytotoxicity from 70-80% to 18-25%, at an E: T ratio of 20:1 (Figure 4A, left). For the consideration of the different proliferative ability between SCCs and control cells, we pretreated tumor cells with Mitomycin C (MMC) for 2 h before cytotoxicity assay. The results were similar to that without MMC pretreatment (Figure 4A, right). To confirm this finding, we analyzed the antitumor effect of DC-CIK cells on HCT116 cells and a similar tendency was seen (Supplementary Figure 6).

We further investigated whether fast-dividing cells were more sensitive to DC-CIK killing by tumor cell proliferation assay. Tumor cells were stained with CFSE and then co-cultured with DC-CIK cells with a Transwell co-culture system. 24 h or 48 h later, tumor cells were collected and used for proliferation assay. We found that proliferation index of control LoVo cells decreased, while that of SCCs was not changed after being treated with DC-CIK cells (Figure 4B). The percentage of CFSE-retaining cells was analyzed 72h after co-cultured with DC-CIK cells by flow cytometry. CFSE-labled LoVo cells cultured for 3 days were seemed as CFSE-negative cells as a negative control. The percentage of CFSE-retaining SCCs was 89.45%. After co-cultured with DC-CIK cells for 3 days, CFSE-positive population of LoVo cells was 58.75%. While that of SCCs was undifferentiated (90.43%) (Figure 4C). In consist with these observations, Ki67 expression in residual LoVo cells treated with DC-CIK cells was lower than that in untreated cells. Ki67 expression in SCCs was not influenced obviously by DC-CIK killing (Figure 4D). These indicated that a distinct subpopulation of slow-dividing cells remained unaffected by DC-CIK killing. Cellular dormancy would imply that a solitary tumor cell in a quiescent state, as defined by an arrest in the G0-G1 phase of the cell cycle [31]. Cell cycle analysis of SCCs revealed increase in G0-G1 phase with only a slight enrichment for the G2/M phase after co-cultured with DC-CIK cells. While cell cycle profiles from LoVo cells after co-cultured with DC-CIK cells showed 2-fold increase in S-phase, suggesting an extended S-phase or arrest (Figure 4E). These data indicated that DC-CIK-based cytotoxicity efficiently eradicated fast-cycling tumor cells *in vitro*, leaving a subpopulation of slow-dividing cells unaffected.

### SCCs enriched by cell cycle inducer combined chemotherapy are more resistant to DC-CIK adoptive immunotherapy *in vivo*

**Figure 5:**
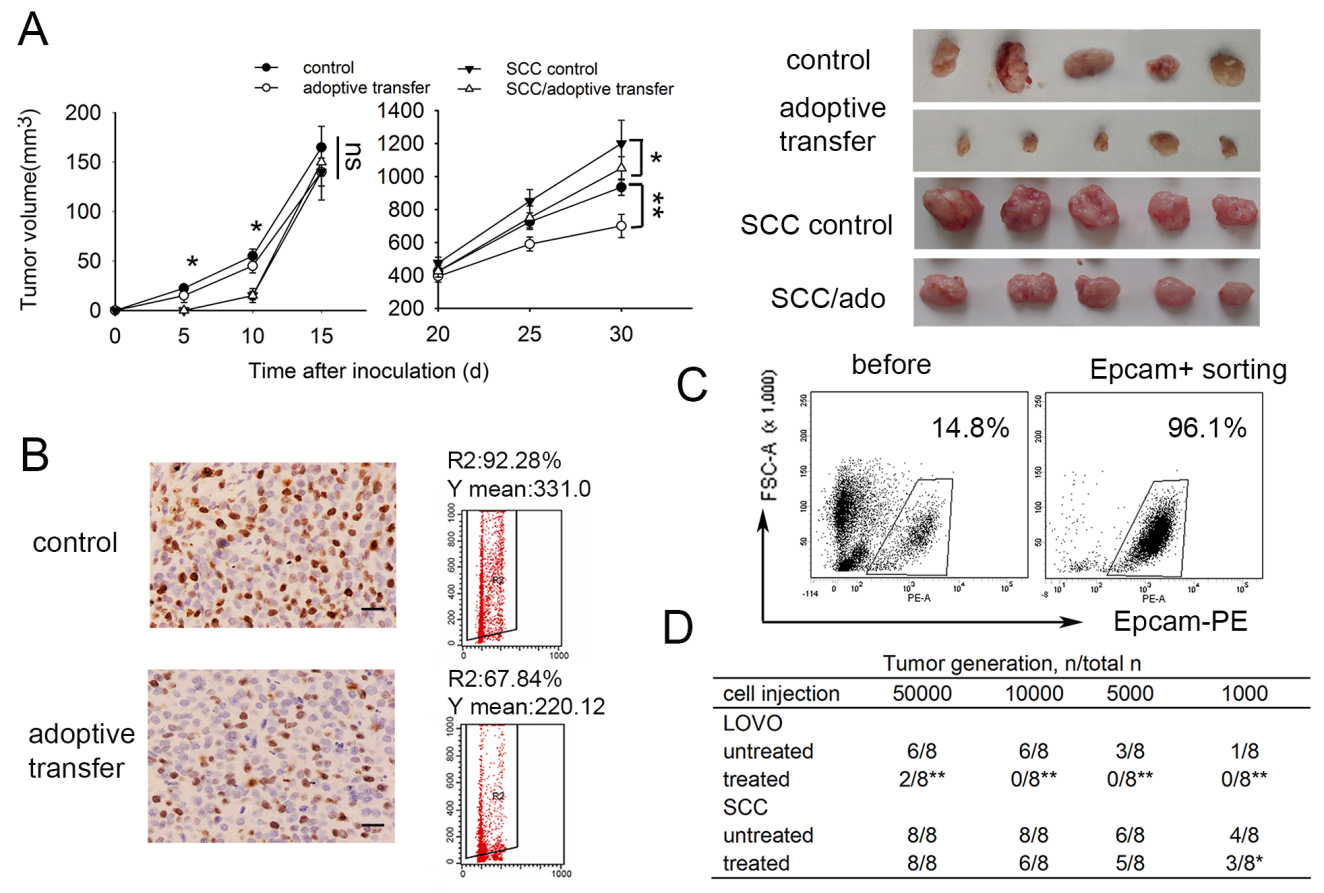
SCCs enriched by cell cycle inducer combined chemotherapy are less sensitive to DC-CIK adoptive immunotherapy *in vivo* **(A)** Nude mice subcutaneously challenged with LoVo cells received adoptive immunotherapy by intravenuous injection of DC-CIK cells. Tumor growth was monitored at indicated time. **(B)** Ki67 staining of xenograft tumor tissues. Ki67 expression in xenograft tumors after adoptive immunotherapy and control tumors were analyzed by immunohistochemisty. Fresh tumor cells prepared from tumor tissues were used for Ki67 staining by flow cytometry. R2 index means percentage of Ki67^+^ cells in tumors. Y mean means mean fluorescence intensity of Ki67^+^ cells. Scale bar represents 50 μm. **(C)** Tumor cells were co-cultured with DC-CIK cells for 48h and then seperated by EpCAM^+^ FACS sorting. Representative images are shown. **(D)** Tumor-generation assay using injection of tumor cells after co-cultured with DC-CIK cells. SCCs or control LoVo cells co-cultured with DC-CIK cells for 48h were sorting as above and then inoculated into Nude mice in a gradient dose. This table presented the result of one experiment. The t-test was carried out to determine the difference between the two groups in three experiments: *P<0.05, **P<0.01.

Previous studies had shown that adoptive DC-CIK cell immunotherapy exerted antitumor effects in Nude mouse model [32]. Here we investigated the effect of DC-CIK based immunotherapy in colorectal carcinoma mouse model. To assess DC-CIK cells infiltrating into tumor, we injected CFSE-labled DC-CIK cells into LoVo colorectal cancer-bearing Nude mice. The fluorescent spots could be observed 24 h after i.v. injection in tumor tissues following by gradually dissipating (Supplementary Figure 7). In consist with previous observation in other study, the delivery of DC-CIK cells were found to inhibit subcutaneous tumor growth in Nude mice challenged with LoVo cells. The delivery of DC-CIK cells had little effect on tumor growth in nude mice challenged with SCCs in early stage of tumor development, and could inhibit tumor growth in later stage slightly (Figure 5A). These may due to the cellular differentiation of SCCs toward proliferation state after a long lagphase *in vivo*. After adoptive immunotherapy, there was a decreased Ki67-expressing in xenograft tumor tissue (Figure 5B), which suggested adoptive immunotherapy may also eliminate fast-dividing cells preferentially *in vivo*.

We wondered whether the increased tumorigenicity of SCCs was less decreased under DC-CIK killing. To do this, we co-cultured SCCs or control LoVo cells with DC-CIK cells for 48 h. Then EpCAM^+^ tumor cells were sorted via fluorescence activated cell sorting (Figure 5C) and inoculated into Nude mice. By injecting 10,000 indicated tumor cells, no tumor formed in 8 mice injected with LoVo cells treated with DC-CIK cells, compared with that in 6 of 8 mice injected with LoVo cells untreated. There is only a modest difference between SCCs and SCCs treated with DC-CIK cells. The similar tendency was observed by injection of others dose of tumor cells (Figure 5D). All these results indicated the increased tumorigenicity of SCCs was less decreased under DC-CIK killing.

### MHC deficiency on SCCs may contributes to resistance to DC-CIK killing

**Figure 6:**
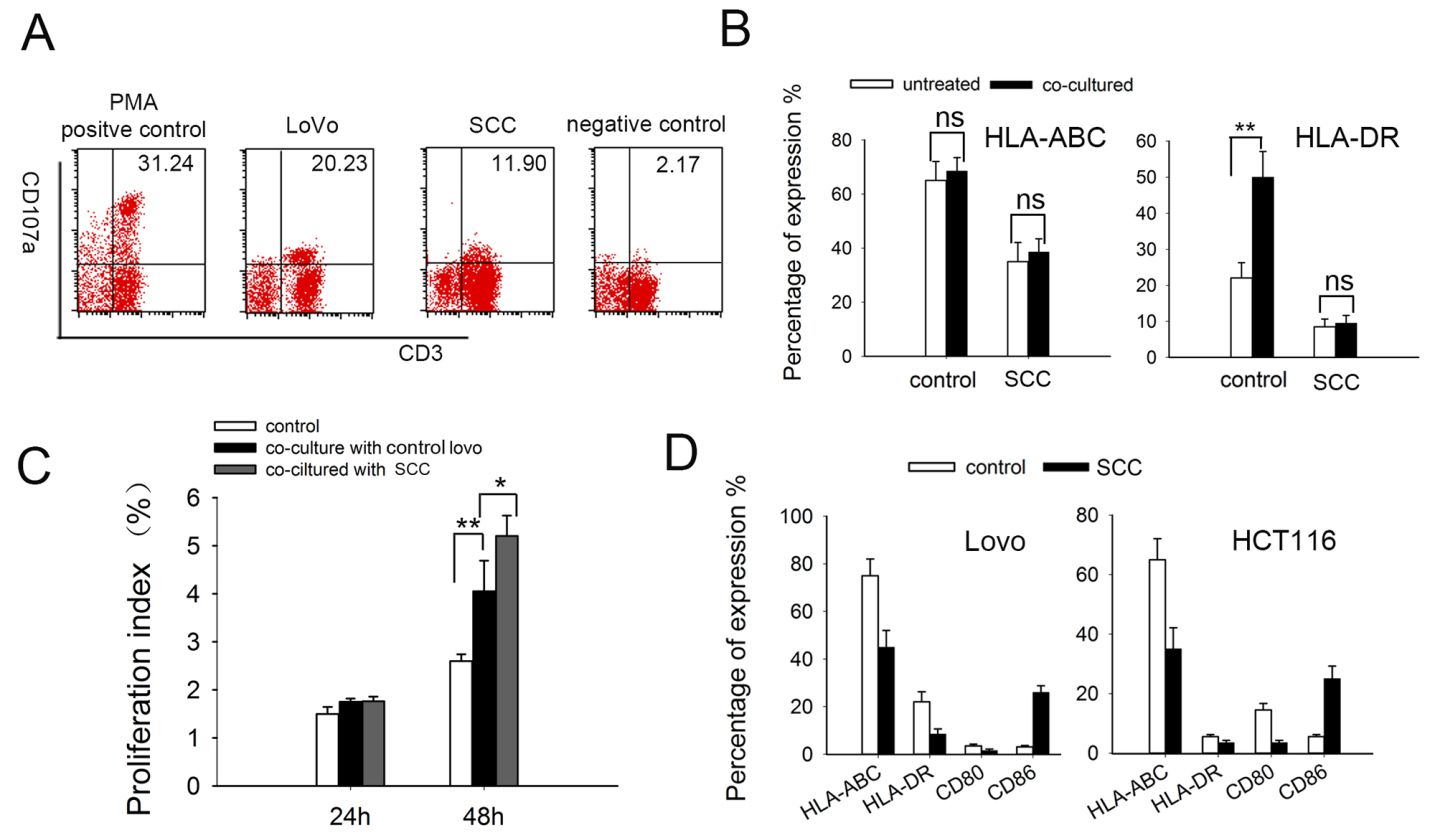
The expressions of immunological markers on DC-CIK and tumor cells **(A)** CD107a degranulation assay on DC-CIK cells. DC-CIK cells were incubated with tumor cells at ratio of 20:1 for 5 hours. CD107a expression was measured by flow cytometry for degranulation of CD3^+^ cells. PMA and ionomycin were added in the positive group and DC-CIK cells alone were used as negative control. **(B)** MHC-I (HLA-ABC)/II (HLA-DR) molecules expression on LoVo cells and SCCs-LoVo after co-cultured with DC-CIK cells. **(C)** DC-CIK cells had significantly higher proliferation after co-cultured with SCCs compared with control LoVo cells, at an effector: target (E: T) ratio of 10:1. Data are pooled from three independent experiments with triplicate samples in each group. **(D)** Expression of MHC and co-stimulatory molecules on the surface of tumor cells. Human colorectal LoVo cells and HCT116 cells were treated with EGF combined with 5-FU. Expression of MHC molecules and CD80/CD86 co-stimulatory molecules were analyzed by flow cytometry. *P* values, *P<0.05, **P<0.01.

Multiple delivering signals were involved in CIK cell activation, resulting in granule exocytosis, cytokine secretion, and cytotoxicity *in vitro* [34, 35]. Here we tested cytotoxic granule release of effector cells using a mAb directed against the lysosomal-associated membrane protein-1 (CD107a) [36, 37]. Consistent with cytotoxicity data, the percentage of CIK cells undergoing degranulation cell was decreased when cocultured with SCCs (Figure 6A). Alteration of MHC expression has been shown to inhibit cytotoxic T-cell mediated lysis in cancer dormancy [38]. Here we found that the expression of MHC class I molecules (HLA-ABC) and MHC class II molecules (HLA-DR) expressed on SCCs were much lower than that on control LoVo cells. Furthermore, expression of HLA-DR on control LoVo cells were much higher after DC-CIK cell killing, which may due to MHC inducing after T-cell activation [39]. Nevertheless, MHC expression on SCCs were not affected (Figure 6B). Lower expression of MHC molecules could be one reason for the resistance of slow-cycling tumor cells to cytotoxic killing.

Interestingly, when DC-CIK cells were stained with CFSE and co-cultured SCCs, the proliferation index was much higher than that co-cultured with control LoVo cells (Figure 6C), implying that SCCs induce increased proliferation of DC-CIK cells. We further investigated co-stimulatory molecules as well as MHC molecules on LoVo and HCT116 cells. The expression of co-stimulatory molecules CD80 was downregulated while CD86 was upregulated in SCCs (enriched from both LoVo cells and HCT116 cells) (Figure 6D), which showed the alteration of immunological markers on SCCs.

## DISCUSSION

In this report, we identified and characterized an intrinsic system for a slow-cycling tumor cell sub-population by cell cycle inducer combined chemotherapy treatment in colorectal cancer cells.

Tumor dormancy has been recognized for many years as a clinical phenomenon in several types of cancer [40, 41]. Tumors in dormancy are mainly constructed of quiescent or slow-cycling tumor cells. Some studies indicated that quiescent stem-like populations might contribute to CSC at least in some tumors [42, 43]. Thus, more research into the identification and biologic character of quiescent or slow-cycling tumor cells is needed.

In the present study, we used EGF as a cell cycle inducer to get more cell-dividing colorectal tumor cells, which were more sensitive to commonly used chemotherapy agent 5-FU *in vivo* and *in vitro*. Our data directly confirm the existence of quiescent like cells after this combined treatment. In the present study, we examined the tumorigenic ability of slow-cycling tumor cells enriched in tumor-bearing mice. We found that the tumorigenic ability of SCCs was much higher than that of control cells, which was in consist with the feature of CSC. Notably, the drug-resistant and tumorigenic ability of SCCs in our model were much higher than that enriched by chemotherapy alone. EGF may contribute to CSC properties, but recent studies showed that EGFR signal was downregulated in advanced CSC [44]. EGFR inhibition induced quiescent CSC in intestinal organoids [22]. SCCs in our model were less sensitive to EGF stimulation. Our combined exposure model may enrich a tumor cell sub-population more approximate to cell-cycle quiescent cells playing a role in CSCs.

In present study, we employed colorectal tumor metastasis by spleen injection. We found that the growth ability of SCCs in spleen was weaker than that of control cells, but its metastasis ability to liver was obviously enhanced. Metastasis involves a cascade of events in which surviving in circulation is involved [45]. Once in circulation, cancer cells must escape the immune system attack and overcome anoikis to extravasate and invade the parenchyma of a distant tissue [46]. Our data in present study showed that the arrest and extravasation of SCCs by *i.v.* injection were higher than that of control cells, which may contribute to the metastasis to distant organs. SCCs survive better in anokis assay and awaked to produce increased MMPs when explored to matrigel (ECM molecules), which is related to higher invasion capacity and higher metastatic potential of tumor cells. It is likely therefore that SCCs in our model were at a alterable state and participate in metastatic dormancy.

Emerging evidence suggests that disseminated dormant tumor cells derived from primary tumors before or after immune surveillance, are responsible for subsequent metastasis [47]. Here we exploited the ability of DC-CIK immune cells to targeted tumor cells. Adoptive transfer of DC-CIK cells (termed as antigen-specific cytotoxic T lymphocytes in some other studies [48]) has already been employed in adoptive immunotherapy [19, 34]. Our present study showed that DC-CIK cells had less significant cytotoxicity against SCCs than fast-dividing cells. Adoptive transfer of DC-CIK cells resulted in a decreased generation of Ki67^+^ proliferating cells, which was in consist with what we observed in *in vitro* experiment.

Three mechanisms are thought to be involved in cancer dormancy: cellular dormancy, angiogenic dormancy, and immune-mediated dormancy [31, 49, 50]. The loss of MHC (major histocompatibility complex) molecule in tumor dormancy allowed them to evade immunosurveillance and increased their intrinsic oncogenic potential [51, 52]. In present study, DC-CIK cells exhibited a lower expression of CD107a and had less potent in cytotoxic activity against SCCs, which may due to the loss of major histocompatibility complex of slow-cycling colorectal cancer cells. Interestingly, SCCs population increased the proliferation of DC-CIK cells, which may due to the overexpression of co-stimulation molecules and SCC-specific antigens.

Recent studies showed that short-term chemotherapy exposure model enriched for a slow-cycling tumor subpopulation. To date, the primary treatment for eliminating slow-cycling tumor cells is to induce them to enter the cell cycle and then kill them using traditional methods [11]. But the characteristics of the remaining cells after cell cycle inducer combined chemotherapy are not well studied. In present study, we showed that the residual cells after the combined treatment were more approximate to tumor quiescence associated with tumor initiating potential. These cells were less sensitive to DC-CIK based immunotherapy, while could promoted their proliferative ability accompanied by the alteration of a series of immuno-molecules. Adoptive cell transfer therapy was a potent universal treatment for cancer [53]. It is likely that specific distinct antigens expressed by CSCs may have distinct antigenicity and thus provide opportunities for enhanced immunotherapy [33]. But there is controversy regarding the identification and isolation of CSCs in different tumors. Our present work should allow for future live enrichment and better characterization of dormancy or slow-cycling subpopulation to develop more targeted therapies.

## MATERIALS AND METHODS

### Ethics statement

Colorectal adenocarcinoma tissue samples were obtained from patients that underwent surgical procedures within the Tongji Hospital of Tongji Medical College, Huazhong University of Science and Technology. Human blood samples were collected from informed donors. Written informed consent was obtained from all research subjects. All protocols were approved by the Ethical Committee of Tongji Hospital, Tongji Medical College, Huazhong University of Science and Technology and were conducted according to the principles of the Declaration of Helsinki. Experimental research involving animals that are reported in the manuscript have been performed with the approval of the Animal Care and Use Committee of Tongji Medical College.

### Animals and cells

BALB/c athymic Nude (nu/nu) mice (6-8 weeks old) were purchased from SLAC Laboratory Animal Co. Ltd. (Shanghai, China). The mice were maintained in the accredited animal facility of Tongji Medical College. Human colorectal cancer cell lines LoVo and HCT116 were purchased from China Center for Type Culture Collection (CCTCC, Wuhan, China) and cultured according to their guidelines. The cell lines were authenticated at China Center for Type Culture Collection (Wuhan, China) in June 2015, using short tandem repeat (STR) DNA profiling (ABI 31300xl Genetic Analyzer; Life Technologies).

### *In vitro* model of SCCs enriched by cell cycle inducer combined chemotherapy

Colorectal cancer cell lines or primary colorectal tumor cells were incubated with human recombinant epidermal growth factor (EGF, 25ng/ml) for 2 d, after which 5-fluorouracil (5-FU) was added for 4 d. Then chemotherapy was removed and new media added. 2 weeks later, the residual tumor cells were harvested for further experiments.

### *In vivo* model of SCCs enriched by cell cycle inducer combined chemotherapy

5×10^5^ primary colorectal tumor cells or LoVo cells were suspended in 50% Matrigel (BD Biosciences) and injected subcutaneously into the flank of Nude mice (20 mice for each cells). The detailed groupings were as follows (Supplementary Figure 3): (1) Group NS, treated with normal saline (NS). (2) Group EGF, treated with subcutaneous EGF administration (25ng/g, once every three days for three times). (3) Group 5-FU, treated with intraperitoneal injection of 5-FU (25μg/g, once every three days for three times) (4) Group EGF+5-FU, treated with EGF administration combined with 5-FU intraperitoneal injection. Tumor growth was monitored every 5 days and the volume of tumor (V) was determined as described previously [54].

### Preparation of single cell suspensions from tumors

Primary colorectal tumors or xenograft tumors were minced completely to the size of 1mm^3^ and then suspended in DMEM/F12 media (Invitrogen, California, USA) containing 1.5mg/ml collagenase IV (Invitrogen, California, USA), 20ug/ml hyaluronidase (Sigma, St. Louis, USA), 1% penicillin/streptomycin (Life technologies, NY, USA) and 1.25mg/ml amphotericin B (Sigma, St. Louis, USA) at 37°C for 1 hour. After digestion, tissues were washed with PBS and filtered through a 40 μm mesh (BD Falcon, CA, USA). To eliminate red blood cells, the cells were incubated in red blood cell lysis buffer (eBioscience, California, USA) on ice for 10 minutes and washed twice with PBS. The cells were then resuspended in PBS for experiments.

### Tumor cell proliferation assay

Tumor cells were labeled with CFSE and cultured away from light. The proliferation of the cells was analyzed by flow cytometry. Flow cytometric analysis included at least 1×10^4^ events on a BD LSR II Flow Cytometer. The proliferation index was calculated in the responder population gate using the ModFit LT for Win32 software. Each sample was assayed in triplicate in three independent experiments.

### Sphere-formation assay

Tumor cells were resuspended in standard sphere-forming medium DMEM/F12 (Invitrogen, California, USA) supplemented with 1× B27 serum substitute (Invitrogen, California, USA), 20ng/ml human recombinant epidermal growth factor and 20ng/ml basic fibroblast growth factor (Sigma, St. Louis, USA). Tumor cells were plated at 200 cells /well in 24-well ultra-low attachment plates (Corning, Massachusetts, USA). For serial sphere-formation assays, the first generation spheres were harvested, disaggregated with 0.025% trypsin/EDTA, filtered through 40 μm mesh and re-plated as above. After 5∼14days, spheres with diameters 50 μm were scored and shown as clonogenicity in the figures.

### Preparation of immune cells

The peripheral blood mononuclear cells (PBMCs) were isolated by Ficoll density gradient centrifugation. The PBMCs were then cultured in fresh serum-free medium (Takara, Japan).

### CIK cells

The PBMCs were cultured in fresh serum-free medium for 2 h. Cells in suspension were transfered to fresh dishes containing serum-free medium with 1,000 U/ml recombinant human IFN-γ at a concentration of 2×10^6^ cells/ml. The next day, immobilized anti-CD3 antibody (2 μg/ml), recombinant human IL-2 (1,000 U/ml) were added to the incubation medium. The medium was replaced by the fresh IL-2 containing medium every 2-3 days up to 7 days. During the generation period, cell number was maintained at approximately 5×10^6^/ml. All the recombinant cytokines were purchased from PeproTech Inc.

### Dendritic cells

Following 2 h of PBMCs incubation in a sterile Petri dish, as previously described, the adherent cells were incubated in serum-free medium containing recombinant human IL-4 (1,000 IU/ml) and GM-CSF (1,000 IU/ml). On day 5, tumor cell lysate (20μg/ml), which was obtained by repeated freezing-thawing, was added to the DCs. On day 6, 100ng/ml tumor necrosis factor-α (TNF-α) was added to the DC culture flask to induce maturation. On day 7, the matured DCs were harvested for phenotypic analysis.

### DC-CIK cells

CIK cells cultured individual for 7 days were harvested and co-cultured with matured DCs at a responder ratio of 3-5:1 for another 2 days, which were used as effector cells for further experiments. These cells were named as DC-CIK cells, which were named cytotoxic T lymphocytes (CTLs) in some other studies [48].

### *In vitro* cytotoxicity assay and transwell co-culture assay

For cytotoxic assay, immune cells were added to different tumors cells at various effector to target ratios (5:1, 10:1 and 20:1) for 48 h. Tumor cells (target) alone or immune cells (effector) alone were used as control. DC-CIK mediated cell cytotoxicity was evaluated using CCK-8 cytotoxicity assay kit according to the manufacturer’s protocol. Cytotoxicity was assessed as follow:

Cytotoxic activity=[1−(A−B)/C]×100%,

Where A is the experimental absorbance from the DC-CIK plus tumor cell co-cultures at 490 nm, B is the absorbance from the DC-CIK alone, and C is the absorbance from the tumor cells alone. Experiments were repeated three times with triplicate samples in each group.

In some experiment, effector cells and target cells were co-cultured with a Transwell co-culture system. Briefly, target cells were plated in 24-well dishes. Effector cells were plated in the Transwell chambers (Corning, NY, USA), and these inserts were placed on top of the wells. The membrane in the Transwell had a 0.4-μm pore size that prevents both cell-cell contact and cell migration but allows the diffusion of soluble factors.

### Animal study

To value the tumorigenesis of SCCs enriched in *in vivo* model, xenograft tumor were dissected and digested 30 days after inoculation. Tumor cells were collected by EpCAM^+^ FACS sorting from single cell suspensions and injected subcutaneously into the flank of Nude mice a gradient dose of 10000, 5000 and 1000 cells (n=8 for each group). The mice were examined visually every day up for 6 weeks. To determine growth potential, tumor cells from xenograft tumor were collected and inoculated to Nude mice in a high dose (5×10^5^ cells/mouse, 5 mice for each group). Tumor growth was monitored once every 5 days up to 30 days.

For adoptive transfer mice model, Nude mice bearing colorectal carcinomas (1 week after being subcutaneous inoculation with tumor cells) received intravenous transfusion of DC-CIK cells (1.5×10^7^ cells per mouse, once every three days for four times). Tumor growth was monitored every 5 days.

To value the tumorigenesis of SCCs enriched in *in vitro* with or without DC-CIK killing, tumor cells were cocultured with DC-CIK cells for 48 h *in vitro*. Then they were isolated by EpCAM+ sorting and injected into the flank of Nude mice at a gradient dose of 1000, 5000, 10000 or 50000 cells. The mice were examined visually every day up to 6 weeks.

For colorectal tumor metastasis model, LoVo cells were transduced with CMV-Fluc-IRES-GFP lentiviral expression vector (GeneChem, shanghai, China). GFP positive cells were enriched through FACS sorting and designated as LoVo-GFP cells. SCCs-GFP cells were enriched from LoVo-GFP cells as described above. Nude mice were inoculated with LoVo-GFP or SCCs-GFP cells by the injection of 1×10^6^ cells into spleen. On day 35 after inoculation, mice were sacrificed and tumor nodes on both spleen and liver were observed and photoed under the stereomicroscope. To assay tumor cell arrest in lung during blood flow, LoVo and SCCs cells were labeled with CFSE, and injected into mice via tail vein (2×10^6^ cells/mouse, n=5 for each group). Lungs of mice were harvested 5 h or 24 h after the injection. Frozen sections were prepared and analyzed by fluorescence microscopy.

All these experiments were repeated three times.

### Flow cytometric analysis

To analyze Ki67 activity, tumor cells were harvested and washed in PBS, then added to 70% ethanol at -20°C overnight. The cells were resuspend in 0.1% Triton-X 100 for 30 min after centrifuge, then washed and incubated with FITC-conjugated mouse-anti-human Ki67 for flow cytometric analysis. For cell cycle analysis, tumor cells were further stained with 100 μl of freshly made PI (propidium iodide) staining solution (PBS, 0.2 mg/ml DNase-free RnaseA and 20mg/ml PI) for 15 min and then analyzed using a FACS Calibur flow cytometer (BD Biosciences). ModFit LT 3.0 software (Verify Software, Topsham, MN, USA) was used to calculate the percentages of cells in each cell cycle phase.

To analyze expression of major histocompatibility complex and costimulatory molecules, fresh tumor cells were stained with mouse anti-human CD80/CD86/HLA-DR antibody for flow cytometric analysis. Parameters were acquired on a FACS Calibur flow cytometer (BD Biosciences) and analyzed with CellQuest software (BD Biosciences). All the Abs were purchased from BD Biosciences. Phenotypes of immune cells were detected by Flow Cytometry as the same method.

CD107a degranulation assay was determined as described previously [34]. Briefly, DC-CIK cells (2 × 10^6^) were stimulated for 5 h in complete medium with 1 × 10^5^ tumor cells. Then brefeldin A (10μg/ml) was added. After 5 hours of stimulation, cells were washed, labeled for 15 minutes at 4°C with anti-CD3 and anti-CD107a mAbs. Cells were analyzed on the CellQuest software.

### Anoikis and FACS analysis

Anoikis was induced as described [55]. Briefly, tumor cells were seeded at a density of 20,000 cells per well (500 μL) in a 24-well ultra-low attachment culture plate (Corning, USA). After 4 days, cells were harvested and resuspended in 500 μL binding buffer supplemented with 5 μL Annexin-V-FITC and 5 μL propidium iodide according to the instruction of Annexin-V-FITC Apoptosis Detection Kit (KeyGEN Biotech, Nanjing, China). The percentage of cells that underwent anokis was defined as the Annexin-V or propidium iodide positive population analyzed on BD FACS Aria II.

### MMP assay by gelatin zymography

Tumor cells were cultured for 48 h in DMEM medium containing 1% FBS in presence or absent of pre-coated matrigel. The assays of MMP-9 and MMP-2 in supernatants were performed as described previously [56].

### Statistics analysis

Results were expressed as mean value ± SD and interpreted by one-way ANOVA. Differences were considered to be statistically significant when *P*<0.05.

## SUPPLEMENTARY MATERIALS FIGURES



## Abbreviations

Pri CRC: primary colorectal tumor cells from patient
SCC: slow-cycling tumor cell
DC: dendritic cell
CIK: cytokine induced killer cell
EGF: epidermal growth factor
EpCAM: epithelial cell adhesion molecule
5-FU: 5-fluorouracil
PBMC: peripheral blood mononuclear cell
APC: antigen presenting cell
ECM: extra-cellular matrix
CSC: cancer stem cell
CFSE: 5, 6- carboxyfluorescein diacetate, succinimidyl ester
MMC: Mitomycin C
MMP: matrix metalloprotein
MHC: major histocompatibility complex
