# Supplementary material for: Characterization and functional analysis of a slow-cycling subpopulation in colorectal cancer enriched by cell cycle inducer combined chemotherapy
Source: Oncotarget. 2017 Jul 26;8(45):78466–79. doi: 10.18632/oncotarget.19638 (PMC5667975; doi:10.18632/oncotarget.19638)
Supplement: Supplementary file 1 [file oncotarget-08-78466-s001.pdf]

Characterization and functional analysis of a slow-cycling subpopulation in colorectal cancer enriched by cell cycle inducer combined chemotherapy

SUPPLEMENTARY MATERIALS

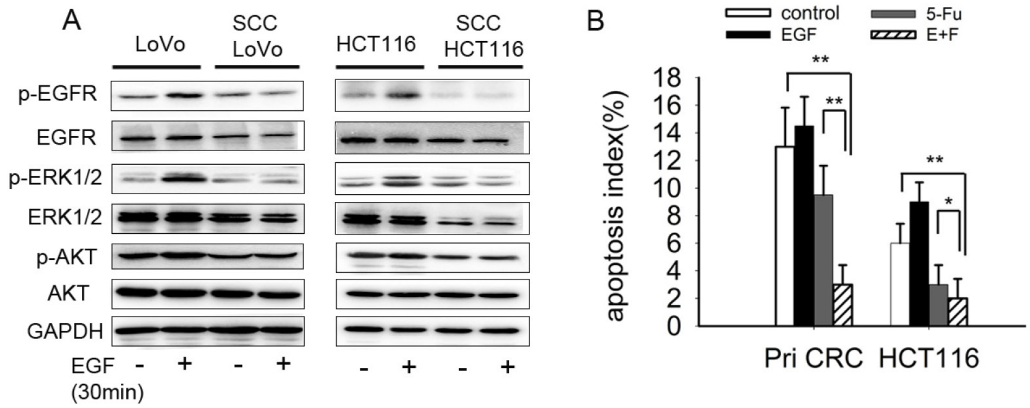

Supplementary Figure 1: (A) EGFR signal in colorectal cancer cells. SCC cells enriched from LoVo and HCT116 cells were incubated with or without EGF (25 ng/ml) for 30 min. Expression of EGFR signal pathway- associated proteins were detected by Western blotting. **(B)** Slow-cycling cells enriched from Pri CRC and HCT116 cells were chemoresistant. Harvested Pri CRC and HCT116 cells after EGF combined 5-FU treatment as well as EGF or 5-FU alone were exposed to 5-FU for another 24 h. 24 h after 5-FU removing, apoptosis of cells was analyzed by flow cytometry. Data are representative of three independent experiments with triplicate samples in each group. *P* values, **P*<0.05; ***P*<0. 01.

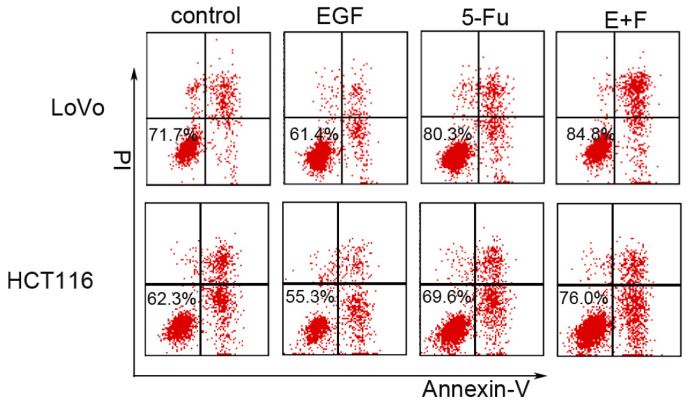

Supplementary Figure 2: SCCs exhibit higher anoikis resistance capacity. Single SCCs and control colorectal cancer cells were seeded at a density of 20,000 cells/well in ultra-low attachment 24-wells. After 4 days anchorage-independent culture, cell apoptosis was evaluated by flow cytometry. The percentage of cells that resistant to anokis was defined as the Annexin-V and propidium iodide negative population.

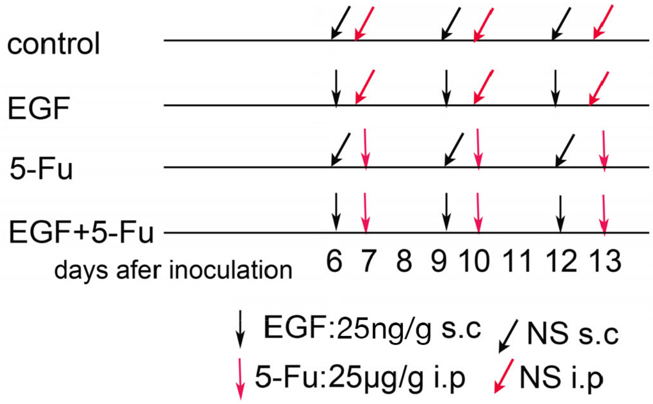

Supplementary Figure 3: Treatment schematic of SCCs enrichment *in vivo*. BALB/C Nude mice were challenged subcutaneously with 5×10^5 colorectal tumor cells on d0 and then treated with the respective regimens according to the treatment schematic. The EGF administration in this study was subcutaneous injection of 25ng/g at d6, d9 and d12. The chemotherapy was intraperitoneal injection of 25μg/g 5-FU at d7, d10 and d13.

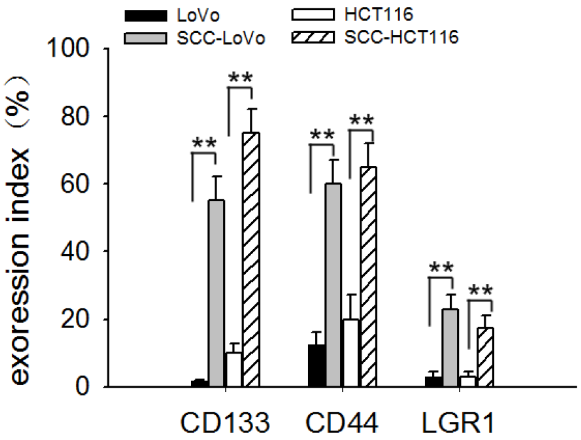

Supplementary Figure 4: CSC markers expressed on SCCs. CSC markers such as CD133, CD44, LGR1 expressed on SCCs and control colorectal cancer cells were analyzed by flow cytometry. *P* values, $**P<0.01$.

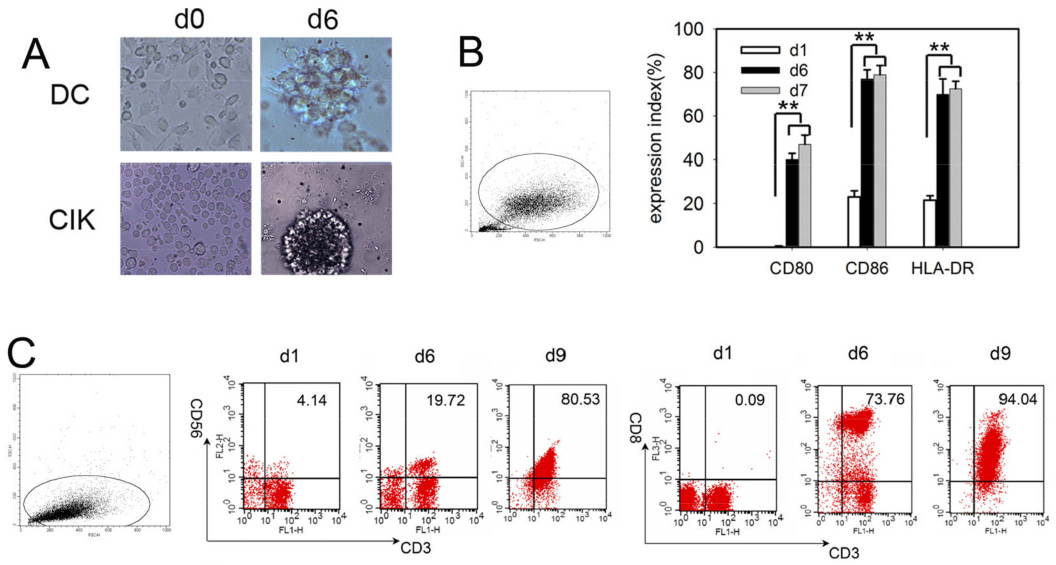

Supplementary Figure 5: Characterization of phenotypes of immune cells. **(A)** DCs or CIK cells cultured for 6 days were imaged. **(B)** DCs cultured for 1day, 6 days and 7 days were harvested and stained for 30 min at 4°Cwith the following monoclonal antibodies: anti-CD80, anti-CD86, or anti-HLA-DR. Then the cells were used for flow cytometry analyse. **(C)** CIK cells cultured for 1day, 6 days and 9 days(co-cultured with matured DCs for another 2 days) were harvested and stained with the following monoclonal antibodies: anti-CD3 and anti-CD56; anti-CD3 and anti-CD8. By flow cytometry, the expression of surface markers on immune cells were examined and recorded. $^{**}P$<0.01.

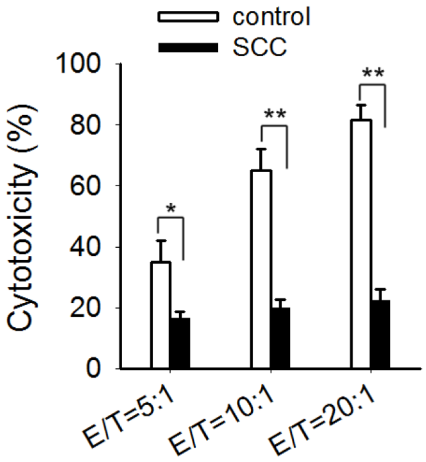

Supplementary Figure 6: Cytotoxic assay of DC-CIK cells on SCCs enriched from HCT116 cells. SCCs or control HCT116 cells were co-cultured with DC-CIK cells for 48h at different E/T radio. The cytotoxicity was measured by CCK-8 assay.

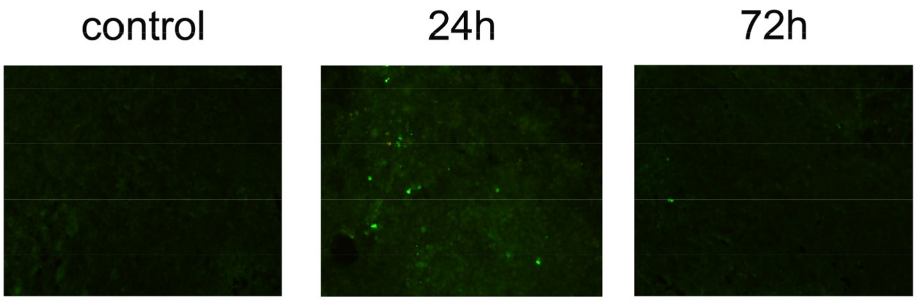

Supplementary Figure 7: Infiltration of immune cells after adoptive transfer. Tumor-bearing Nude mice received adoptive transfer by intravenous injection of CFSE-labled DC-CIK cells. Tumor tissues were harvested 24h or 72h after injection. Frozen sections were prepared. Infiltration of immune cells in tumor tissue was analyzed by fluorescence microscopy.
